# Management of Greater Trochanteric Pain Syndrome: A Narrative Review

**DOI:** 10.7759/cureus.85859

**Published:** 2025-06-12

**Authors:** Jaskiran K Gill, Gregory Neal-Smith, Abdel Saed, Amjad Burgan, Scott Fernquest

**Affiliations:** 1 Trauma and Orthopedics, Queen Alexandra Hospital, Portsmouth, GBR; 2 Trauma and Orthopedics, Poole Hospital, Poole, GBR; 3 Orthopedics, Barts Health NHS Trust, London, GBR; 4 Trauma and Orthopedics, University Hospitals Bristol and Weston NHS Foundation Trust, Bristol, GBR; 5 Trauma and Orthopedics, Gloucester Royal Hospital, Gloucester, GBR

**Keywords:** gluteal tendinopathy, greater trochanteric pain syndrome, gtps, lateral hip pain, trochanteric bursitis

## Abstract

Greater trochanteric pain syndrome (GTPS) is characterized by pain and tenderness over the lateral aspect of the hip. It encompasses a spectrum of conditions, including abductor tendinopathy, trochanteric bursitis, and external coxa saltans. Following diagnosis, managing GTPS can be challenging due to the variability in pathology and the wide range of available treatments, many of which are supported by low levels of evidence. At present, there is no clear consensus on the optimal management approach for this condition. This review evaluates the existing literature on GTPS management, aiming to provide clinicians with a framework to guide treatment decisions. Articles were sourced from PubMed and MEDLINE using the terms “greater trochanteric pain syndrome”, “trochanteric bursitis”, and “gluteal tendinopathy”. Findings suggest that targeted physiotherapy (PT) offers superior long-term outcomes compared to shock wave therapy and corticosteroid injections (CSI). In one study, 60.5% of patients reported symptom resolution at 15 months. Extracorporeal shock wave therapy (ESWT) demonstrated a 68.3% improvement in Visual Analogue Scale scores compared to control groups. Combining PT with CSI appears to be more effective in managing acute symptoms than PT alone. The evidence supporting platelet-rich plasma (PRP) remains inconclusive, with ongoing research needed to clarify its role. Surgical intervention is considered when conservative and medical treatments have failed, with various operative techniques showing improvements in pain scores over time. In conclusion, PT and home exercise programs have shown long-term benefits for most patients, although improvements may not be immediate. CSIs can offer short-term relief but are most effective when combined with PT for sustained benefit. Focused ESWT has limited but promising evidence supporting its use, with a low risk of adverse effects. Although PRP has shown potential in some studies, further research is necessary before it can be recommended as part of standard treatment protocols.

## Introduction and background

Greater trochanteric pain syndrome (GTPS) is characterized by pain and tenderness over the lateral aspect of the affected hip [1], typically reproduced by lateral pressure over the greater trochanter (GT), weight-bearing, or resisted abduction [2,3]. The term encompasses a range of conditions affecting the peritrochanteric space. The most common cause of GTPS is abductor tendon pathology, which accounts for 15-50% of cases [4]. Other contributing factors include trochanteric bursitis, coxa saltans externa, and abnormalities of the iliotibial band (ITB) [5-7]. The condition is believed to result from repetitive friction between the ITB and the GT and from repeated microtrauma to the muscles attaching to the GT [8]. This process can lead to tendon degeneration, inflammation, and increased tension in the ITB [8]. The heterogeneous nature of GTPS often complicates accurate diagnosis and management.

GTPS is a common condition, with an incidence of 1.8 per 1,000 adults annually in the primary care setting [5]. Although it can occur across all populations, it shows a female predominance of 2-3:1 and is most frequently seen in individuals in their 40s to 60s [5]. Diagnosis is primarily clinical; however, imaging is often used to rule out other causes of lateral hip pain, such as osteoarthritis, femoroacetabular impingement, lumbar spondylosis, hernias, and avascular necrosis of the femoral head [5,9].

Following diagnosis, the management of GTPS can be challenging due to the variety of available treatment modalities and the limited strength of supporting evidence. Currently, there is no consensus on the optimal treatment approach [4]. Available treatment options include physiotherapy (PT), nonsteroidal anti-inflammatory drugs, corticosteroid injections (CSIs), radial and focused shockwave therapy, and platelet-rich plasma (PRP) injections [10]. While most patients respond to conservative management, those with persistent symptoms may benefit from surgical intervention [8].

The primary aim of this review was to critically evaluate the existing published evidence on the management of GTPS to support clinical decision-making. Specifically, the review sought to identify, synthesize, and appraise the range of treatment modalities currently available, including conservative, pharmacological, interventional, and surgical approaches.

The objectives of this review were to (1) categorize the available treatment modalities according to the strength and quality of supporting evidence; (2) assess the clinical effectiveness of individual therapies and combination treatment strategies; (3) highlight areas of consensus and inconsistencies within the literature; and (4) provide a practical, evidence-based framework that clinicians can use to tailor patient management.

## Review

Methods 

In May 2022, a comprehensive search of PubMed and MEDLINE was conducted. The following Medical Subject Headings (MeSH) terms and keywords were used: “greater trochanteric pain syndrome”, “trochanteric bursitis”, and “gluteal tendinopathy”. These terms were combined using Boolean operators (AND/OR). The search was limited to articles published in English-language journals, with no date restrictions applied. Duplicate articles were removed, and the remaining studies were screened by title and abstract, followed by a full-text review.

This review was conducted using the PICOS framework: Population (P): Adults (>18 years) diagnosed with GTPS, including those with gluteal tendinopathy and trochanteric bursitis. Studies involving both male and female participants were included, focusing on patients presenting with non-traumatic lateral hip pain. Intervention (I): Any therapeutic intervention aimed at managing GTPS, including but not limited to PT, CSIs, PRP, shockwave therapy, surgical procedures, and multimodal approaches. Comparison (C): Most studies compared interventions against placebo, sham treatments, no treatment, or alternative therapies. However, as this was a narrative review, not all included studies had comparator groups. Outcomes (O): Primary outcomes of interest were patient-reported outcome measures, pain reduction, functional improvement, and recurrence rates. Secondary outcomes included adverse events and the duration of treatment efficacy. Study design (S): Only studies focused on the management of GTPS were included. Exclusion criteria were studies solely investigating diagnosis, case reports, and small case series (n < 10), and publications that had been superseded by higher-quality research.

Results

Nonoperative Management

Nonoperative intervention remains both the first-line and the cornerstone of treatment for GTPS. Published reviews support a combination of rest, ice, heat, weight loss, activity modification, and PT [5].

PT and home exercise: Home-based training programs, guided by physiotherapists, are the initial recommended treatment for GTPS. The most widely adopted protocol, developed by Rompe et al., involves slow, repetitive movements performed over a 12-week period [10]. The program includes piriformis and standing ITB stretches, straight leg raises, wall squats with a ball, and gluteal strengthening exercises. All participants received a practical session with a physiotherapist and written instructions to continue at home. They were advised to perform the routine twice daily during the first week, then once daily for the next 12 weeks. Follow-up visits with a clinician at weeks two and four assessed adherence. By week six, patients were encouraged to gradually resume their previous sporting activities.

Outcomes assessed via the Likert Scale Rating showed superior long-term results compared to other evidence-based interventions, such as shock wave therapy and CSIs. At 15 months, 60.5% of patients reported complete resolution of GTPS following the home program. Data also showed a positive correlation between program duration and improved patient-reported outcomes. Although patients receiving shockwave therapy experienced longer symptomatic relief than those given CSIs, the latter group frequently reported symptom recurrence over time [10].

A more recent review by Mellor et al. supported these findings, showing that education and exercise programs led to greater symptom relief at eight and 52 weeks compared to both CSIs and watchful waiting (WS) [6,11]. Their intervention included 14 PT sessions over eight weeks combined with a home exercise program.

Focused extracorporeal shock wave therapy (ESWT): ESWT, particularly when combined with eccentric exercise, has emerged as a promising treatment for GTPS. In a study by Shi et al., 53 patients were randomly assigned to a control group (24 patients) or an observation group (29 patients) [12]. At one month, no significant differences were seen in Harris Hip Score (HHS) and Visual Analogue Scale (VAS) scores. By two months, VAS scores improved more in the observation group (58.62%) compared to the control group (29.16%), although HHS remained similar. At six months, both VAS and HHS had significantly improved in the ESWT group.

Ramon et al. also demonstrated that ESWT improved VAS scores by 68.3% at two months, compared to 35.4% in the control group. At six months, significant clinical and functional improvements persisted in the ESWT group [13].

According to Rompe et al., improvements in both HHS and VAS scores were sustained up to 15 months post-treatment, with 30.7% of patients reporting complete resolution of symptoms, compared to just 1.3% at one month [10]. In a prospective study by Heaver et al., 80% of patients tested negative on the Trendelenburg test post-treatment, compared to 20% at baseline (p < 0.001) [14].

CSI: The effectiveness of CSIs for GTPS varies widely across studies. Most data involve single injections, although Lustenberger et al. found that 33% of patients required a second injection, and in rare cases, up to five were needed [15].

When combined with PT, CSI has been shown to relieve acute symptoms more effectively than PT alone. In a randomized controlled trial (RCT) by Brinks et al., 120 patients were assigned to receive either analgesia and PT or the same regimen with an added CSI [16]. A second injection was allowed between three weeks and three months after the first. At three months, 55% of patients in the CSI group reported full or substantial recovery, compared to 34% in the non-CSI group. However, by 12 months, recovery rates were similar (CSI vs. non-CSI: 61% vs. 60%).

Nurkovic et al. also reported better outcomes in patients who received PT following CSI compared to those who received CSI alone. In a retrospective observational study, 49% of patients improved with combination therapy versus 39% with CSI alone [17]. These studies confirm CSI’s short-term efficacy. Moreover, Jarlborg et al. found that patients who experienced >50% pain reduction within 30 minutes of injection were more likely to report sustained improvements [18].

PRP: The evidence supporting PRP for GTPS remains inconclusive. However, a double-blind RCT by Fitzpatrick et al. compared PRP with CSI for the treatment of gluteal tendinopathy and reported an 82% improvement in patient-reported outcomes in the PRP group versus 56.7% in the corticosteroid group at 12-week follow-up [19].

A 30-patient RCT conducted by Jacobson et al. compared ultrasound-guided PRP injection with percutaneous tendon fenestration [20]. In this study, the fenestration group received 20-30 needle passes, while the PRP group received only 10. Patient-reported outcomes revealed that the fenestration group experienced better results in the early phase. At baseline, one week, and two weeks post-treatment, outcome scores assessing pain and sleep disruption were 32.4, 16.8, and 15.2 for the fenestration group, compared to 31.4, 25.5, and 19.4 for the PRP group, indicating greater early improvement in the fenestration group. However, by three months, the PRP group reported greater improvement (79%) compared to the fenestration group (71%). Although this difference did not reach statistical significance, both treatments were considered largely effective [1,20].

In another RCT, Begkas et al. compared PRP with CSI. At one-month follow-up, the CSI group showed significantly better results in pain and function: the HHS improved by 32.81 in the CSI group versus 24.92 in the PRP group, while the VAS improved by 4.59 versus 2.57, respectively [21]. However, at the two-year follow-up, the CSI group’s scores had returned to pre-injection levels, whereas the PRP group continued to show improvement, with HHS and VAS increases of 40 and 5.7, respectively [21].

A 102-patient double-blind RCT by Oderuth et al., investigating PRP versus placebo with a 12-month follow-up, is currently underway, but results have not yet been published [22].

Simple multimodal approach: A three-month regimen combining rest, ice, heat, PT, ESWT, and CSIs enabled 83% of patients to return to their previous labor-intensive occupations and 66% to resume previous levels of sporting activity [15].

Operative Management

Surgical intervention for GTPS is reserved for cases where conservative and medical therapies have failed. Diagnostic imaging showing bursitis or tendon pathology is typically required to support the decision for surgery. The choice of operative procedure depends on the type and severity of the pathology.

In a study by Drummond et al., 49 patients with treatment-resistant GTPS underwent endoscopic ITB release and trochanteric bursectomy [23]. Intraoperative evaluation noted gluteal tendon integrity, with tendon damage observed in eight cases. Patients were followed up for over 20 months. At final follow-up, 24.6% of patients reported no pain, 38.6% experienced very mild pain (VAS: 1-2), 29.8% reported mild to moderate pain (VAS: 3-7), and 7% continued to experience severe pain (VAS: 8-10). Overall, the mean VAS score improved from 7.8 to 2.8.

In a prospective single-center study, patients requiring operative treatment were classified using the Lall GTPS classification system [4]. Surgical interventions were selected according to classification type. VAS scores were recorded preoperatively and at two years postoperatively, with all interventions resulting in significant pain reduction (Table 1). These findings suggest that, when used judiciously, operative treatment can be an effective option for managing GTPS.

**Table 1:** VAS scores for different surgical interventions for the treatment of GTPS GTPS, greater trochanteric pain syndrome; VAS, Visual Analogue Scale Adapted from Annin et al. [4]

GTPS etiology	Surgical intervention	Number of patients	Pre-op VAS	Post-op VAS
Isolated trochanteric bursitis	Trochanteric bursectomy	109	5.52	2.35
Trochanteric bursitis with gluteus medius surface fraying	Trochanteric bursectomy with micropuncturing	29	5.39	1.82
Partial-thickness gluteus medius tear (<25%)	Endoscopic repair with suture staple	20	6.09	1.99
Partial-thickness gluteus medius tear (>25%)	Trans-tendinous repair	118	5.68	2.03
Full-thickness gluteus medius tear	Endoscopic double-row suture repair	31	5.91	2.1
Full-thickness gluteus medius tear	Open double-row suture repair	13	3.67	1.11
Full-thickness retracted tear	Gluteus maximus/tensor fascia lata transfer	7	4.82	0.55

Discussion 

GTPS is a complex condition, both in terms of its etiology and its management. Currently, there is no established consensus on the optimal treatment strategy. The aim of this review was to evaluate the available evidence and propose a management framework for clinicians.

PT and Home Exercise 

PT and home exercise represent reasonable first-line treatments for patients with GTPS. The studies reviewed demonstrate that structured rehabilitation programs lead to long-term symptom improvement in most patients, without the risks associated with CSIs or the cost of ESWT. However, the benefits of PT are not immediate, and adjunctive therapies may be necessary to alleviate acute pain and enable patient participation in rehabilitation programs. For instance, CSI may provide short-term symptomatic relief that facilitates engagement in PT. For patients who fail to respond to PT or experience symptom relapse, further treatment options should be considered [5,10,11].

The efficacy of structured PT in managing GTPS continues to be studied. Almousa et al. are planning an RCT comparing PT to usual care. Patients in the intervention arm will participate in a structured educational program and attend six face-to-face PT sessions. The control arm will receive a leaflet promoting physical activity and reassurance about the self-limiting nature of GTPS [24]. This study may provide a clearer treatment framework and more up-to-date evidence regarding the effectiveness of PT in GTPS.

ESWT

ESWT has emerged as an effective treatment modality, with evidence supporting its long-term (>12 months) benefit in pain reduction. Similar to PT, ESWT does not typically provide immediate relief; however, patient-reported improvements are observed around two months after treatment [10]. Ramon et al. conducted a particularly valuable study in which patients were randomized to receive either ESWT or sham ESWT, both alongside PT [13]. This design offers robust evidence for the combined efficacy of ESWT and PT in GTPS management.

This finding is supported by two systematic reviews - by Rhim et al. and Harding et al., both of which reported improvements in GTPS with shock wave therapy compared to control groups. However, both reviews also emphasized the need for higher-quality RCTs to provide more definitive conclusions [25,26]. Rhim et al. included eight RCTs (754 patients) and found significant short-term pain improvements in the SWT group, as well as functional improvements at six months. Nonetheless, seven of the eight trials were at high risk of bias, limiting the strength of their findings [25]. Harding et al. included 12 studies (1,121 participants) and found improvements in the low-quality evidence studies, but no statistically significant results in those with moderate-quality evidence. Despite this, the authors noted the low incidence of side effects associated with SWT, suggesting it remains a viable treatment option [26].

CSI

CSI remains the most effective short-term treatment for GTPS [10,11]. Its benefits, however, are often limited to the acute phase, and many patients relapse if no other treatment is initiated. Identifying patients unlikely to benefit from CSI is crucial to avoid prolonged disability. Jarlborg et al., in a small study of 44 patients, used symptomatic improvement within 30 minutes of injection to predict longer-term outcomes. They found that those with >50% VAS score improvement within 30 minutes had the most favorable long-term results [18].

PRP

The role of PRP in GTPS management remains uncertain, but it may be most applicable to chronic cases [1,21]. Fitzpatrick et al. conducted a high-quality double-blind RCT (JADAD score: 5) comparing PRP with CSI and evaluated both short- and long-term outcomes. The study was well-reported and included 40 patients, although its findings would be more robust with a larger cohort [19].

Similar observations were made by Jacobson et al., who found that percutaneous tendon fenestration offered better short-term outcomes than PRP, although PRP outperformed fenestration at three months [20]. This study, however, included only 15 patients and had a limited follow-up period. Begkas et al. also conducted a small RCT (n = 24), which yielded encouraging results for PRP but again highlighted the need for studies with larger sample sizes [21].

A significant limitation in evaluating PRP’s efficacy lies in the lack of standardization. Mahajan et al. performed a systematic review of four eligible RCTs, two of which showed significant improvements in pain and function with PRP, while the other two did not. Differences in PRP concentration, injection sites, and volumes contributed to this variability. The authors concluded that standardized protocols are necessary for future RCTs to determine PRP’s true efficacy in GTPS [27].

Operative Management

When imaging identifies a specific etiology, surgical intervention can be effective and should be considered. Both endoscopic and open procedures have been shown to reduce pain, as evidenced by the study conducted by Annin et al. [4]. Their simple, algorithm-based approach proved effective in a large cohort of 324 patients, with a minimum two-year follow-up providing valuable long-term outcome data. Nonetheless, surgical treatment carries inherent risks, such as infection and neurovascular injury, and requires specialized resources. Therefore, operative management should be reserved as a final option for treatment-resistant GTPS once all conservative and minimally invasive measures have failed [4].

Emerging therapies may expand the treatment options available in the future. For instance, Yerra et al. conducted a single-center retrospective study involving 68 patients undergoing percutaneous ultrasound tenotomy for refractory GTPS. Patients showed statistically significant improvements in pain and function after one year. However, the lack of a control group limits the generalizability of these results, and further RCTs are needed to validate the treatment’s effectiveness [28].

Limitations

Due to the absence of a standardized treatment algorithm for GTPS, the conclusions in this review are based on existing literature rather than uniform trials. Moreover, the current body of evidence is limited by the small size and low quality of many studies. Another limitation is the exclusion of non-English studies, which may have led to the omission of valuable and potentially contradictory findings. To develop formal guidelines or treatment algorithms, large-scale RCTs with standardized outcome measures are urgently needed.

## Conclusions

There is limited high-quality published data on the management of GTPS; however, conclusions drawn from the available literature may still hold valuable clinical relevance. The findings from this review suggest a potential treatment algorithm that could guide future interventional studies in GTPS. Based on current evidence, we propose that PT and home exercise should serve as the first-line management for patients diagnosed with GTPS. This approach is relatively inexpensive, low risk, reproducible, and effective regardless of the underlying etiology. For patients experiencing acute pain that hinders participation in rehabilitation, CSI may be used to provide short-term symptomatic relief. However, CSI should always be administered in conjunction with PT, as it offers minimal long-term benefit when used alone. If pain persists despite CSI, further injections are not recommended, and patients should instead be referred for additional investigation to determine the underlying cause of their GTPS.

For patients with chronic or refractory GTPS, ESWT may be considered alongside PT. Patients should be informed that while ESWT has been shown to provide long-term symptom relief, it has a limited impact in the acute setting. The role of PRP remains uncertain, and more robust data are needed before it can be confidently included in standard treatment pathways. Operative interventions should be reserved for cases of persistent, debilitating pain where all other treatment options have been exhausted and a clear etiology has been identified. In such cases, surgery can be effective when performed within a specialist service, ensuring accurate diagnosis, appropriate patient selection, and optimal delivery of care.
